# Malignant Pleural Effusion: Diagnosis and Treatment—Up-to-Date Perspective

**DOI:** 10.3390/curroncol31110507

**Published:** 2024-11-02

**Authors:** Riccardo Orlandi, Andrea Cara, Enrico Mario Cassina, Sara Degiovanni, Lidia Libretti, Emanuele Pirondini, Federico Raveglia, Antonio Tuoro, Sara Vaquer, Stefania Rizzo, Francesco Petrella

**Affiliations:** 1 Department of Thoracic Surgery, Fondazione IRCCS San Gerardo dei Tintori, 20900 Monza, Italy; riccardo.orlandi@unimi.it (R.O.); andrea.cara@irccs-sangerardo.it (A.C.); enricomario.cassina@irccs-sangerardo.it (E.M.C.); sara.degiovanni@unimi.it (S.D.); lidia.libretti@irccs-sangerardo.it (L.L.); emanuele.pirondini@irccs-sangerardo.it (E.P.); federico.raveglia@irccs-sangerardo.it (F.R.); antonio.tuoro@irccs-sangerardo.it (A.T.); sara.vaquer@unimi.it (S.V.); 2 Imaging Institute of Italian Switzerland (IIMSI), Ente Ospedaliero Cantonale, Via Tesserete 46, 6900 Lugano, Switzerland; stefania.rizzo@eoc.ch; 3 Facoltà di Scienze Biomediche, Università della Svizzera Italiana, Via G. Buffi 13, 6900 Lugano, Switzerland

**Keywords:** malignant pleural effusion, pleural fluid, pleural biopsy, thoracoscopy, talc poudrage, indwelling pleural catheter

## Abstract

Malignant pleural effusion is the presence of malignant cells within the pleural fluid, representing the second most common cause of pleural exudate. Although diagnostic methods and management techniques for malignant pleural effusion have dramatically improved over the decades, the current treatment is still palliative, aiming to remove pleural fluid, possibly prevent its recurrence, and alleviate symptoms through a wide range of available procedures. Treatment should be tailored to the individual patient, considering comorbidities, size of the effusion, rate of fluid accumulation, underlying cardiac or respiratory conditions, rate of recurrence, presence of loculations or trapped lung, tumor characteristics, cancer type, and patient preferences. This manuscript aims to review the available literature and to present the latest evidence on malignant pleural effusion management in order to provide an updated perspective on its diagnosis and treatment.

## 1. Introduction

Malignant pleural effusion (MPE) is the presence of malignant cells within the pleural fluid (PF) [1]. Accumulation of MPE within the pleural space is due to impairments in the Starling forces controlling fluid biomechanics by cancer cells [2], leading to increased production or decreased reabsorption of fluid. MPE is the second most common cause of pleural exudate [3]. Approximately 15–20% of cancer patients experience MPE, with an estimated combined incidence of 500,000 cases in the USA and Europe [4]. Its global incidence is estimated to be 70 per 100,000 persons [5], representing a non-negligible burden on global healthcare systems. Most MPE cases are symptomatic, frequently presenting with dyspnea [6]; other symptoms may include chest pain, a persistent dry cough, or a sensation of early fullness due to pressure from effusion on the stomach.

The detection of malignant cells in the PF indicates advanced or widespread cancer, which typically corresponds with a reduced life expectancy: lung cancer patients with MPE have an average survival of 5.5 months [7], while survival for all cancers ranges from 3 to 12 months depending on the tumor type and comorbidities [8]. Two prognostic scores have been validated in MPE, the PROMISE and the LENT scores [9,10], based on biological and clinical variables.

Although diagnostic methods and management techniques for MPE have dramatically improved over the decades, the current treatment is still palliative [11], aiming to remove PF, possibly prevent its recurrence, and alleviate symptoms through a wide range of available procedures including repeated thoracentesis, chest tube positioning, medical or surgical thoracoscopy, talc poudrage or slurry, and indwelling pleural catheter (IPC).

Treatment should be tailored to the individual patient, considering comorbidities, size of the effusion, rate of fluid accumulation, underlying cardiac or respiratory conditions, rate of recurrence, the presence of loculations or trapped lung, tumor characteristics, cancer type, and patient preferences.

This manuscript aims to review the available literature and to present the latest evidence on MPE management in order to provide an updated perspective on its diagnosis and treatment.

## 2. Diagnosis

**Figure 1:**
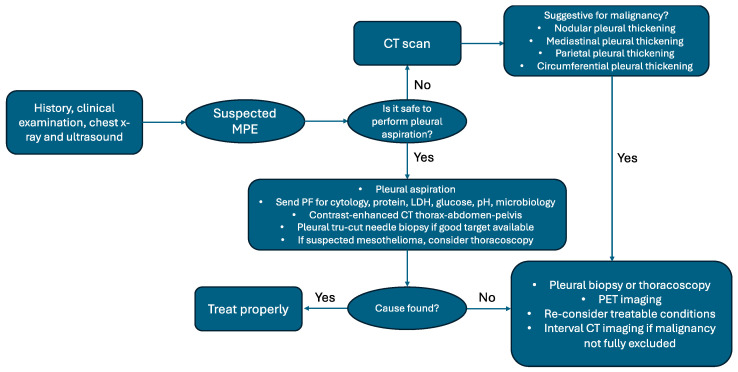
Diagnostic algorithm in MPE (adapted from [14]).

When MPE is suspected, thorough physical examination and clinical assessment are crucial in guiding the subsequent diagnostic pathway. Imaging is essential in diagnosing pleural effusion, assessing its extent, identifying underlying malignancies, and guiding or planning invasive procedures. In this scenario, the chest X-Ray remains a valuable and quickly accessible diagnostic tool. Nevertheless, no single test is definitive on its own. In recent years, there have been notable advancements in the diagnosis of pleural diseases, particularly with the introduction of thoracic ultrasound [12], which has become a key component in evaluating pleural conditions, including MPE. Given that initial pleural fluid aspiration often has low sensitivity in diagnosing MPE, a biopsy for histological examination of pleural tissue is typically needed to confirm the diagnosis and guide cancer treatment. However, there are instances where pleural fluid alone suffices for diagnosis and molecular testing of malignancy, occurring in about 30% of cases [13] (Figure 1).

### 2.1. Imaging Tests

#### 2.1.1. Chest X-Rays

Posteroanterior chest X-Rays are typically the initial diagnostic test performed to assess the signs and symptoms of pleural effusion in patients with cancer [15]. Pleural effusion (PE) can be radiographically detected when the fluid volume reaches 200 mL, with chest X-Rays being able to identify as little as 50 mL of PF in lateral views [16]. Effusions with a fluid volume of less than 500 mL, which occur in approximately 10–15% of cases, are generally not symptomatic. Radiographic indicators such as blunting of the costophrenic angle, shifting of the mediastinum, rib stretching, and lowering of the hemidiaphragm can suggest a diagnosis. A massive effusion, which fills an entire hemithorax, is often associated with a mediastinal shift and inversion of the diaphragm. The occurrence of a massive effusion, fluid loculation, and reduced lung volume on the same side as the effusion raises the suspicion of a malignant cause [17]. Additionally, a mass may be visible if there is a primary lung tumor, while prominence of the hilum might indicate a central tumor or lymph node enlargement.

#### 2.1.2. Thoracic Ultrasonography

Thoracic ultrasound is more sensitive than chest X-Rays for detecting pleural effusion [18]. It can identify smaller fluid volumes and assist in guiding both diagnostic and therapeutic procedures [19]. Using transducer probes with frequencies between 3.5 and 5 MHz allows for good penetration and optimal spatial resolution, making it possible to detect septations (loculated fluid collections), hemothorax, and organized fluid collections. Ultrasound can also differentiate between pleural effusion, lung consolidation, and pleural thickening [20]. Pleural metastases may appear as small, lenticular hypoechoic masses close to the chest wall or as complex echogenic masses [21].

Preprocedural ultrasound is effective in identifying the optimal site for thoracentesis or chest drain positioning. A grading system for pleural effusion severity on ultrasound assessment has been proposed [22], based on anatomical extent, radiological landmarks, and the number of intercostal spaces involved: extensive pleural effusion is characterized by partial displacement of the upper lung lobe, atelectasis of the lower lobe or partial upper lobe atelectasis, and the involvement of three to four intercostal spaces; massive pleural effusion involves complete lung collapse, atelectasis of the entire lung with visible hilum, and affects four or more intercostal spaces. The use of thoracic ultrasound has been shown to reduce the risk of complications like hemothorax and pneumothorax following thoracentesis [23] and can rapidly detect post-procedural complications: lung motion, seen as pleural sliding on ultrasound, may be absent if a post-procedure pneumothorax occurs.

#### 2.1.3. Computed Tomography Scan

Computed tomography (CT) scans have high specificity but low sensitivity in distinguishing benign pleural effusion from MPE and in differentiating mesothelioma from pleural metastasis [24]. According to Hallifax and colleagues [25], CT identified MPE with a sensitivity of 68% and a specificity of 78%. The positive predictive value for identifying MPE was 80%, while the negative predictive value was 65%, indicating that about one-third of patients suspected of having MPE may still have it despite a negative CT scan. Dual-energy spectral CT scans seem to increase accuracy in differentiating malignant from benign pleural disease [26].

Mediastinal lymph node involvement and related parenchymal abnormalities are often better detected with CT, which is considered the gold standard for screening patients with suspected pleural malignancy [27], preferentially when contrast enhanced. Indicators on a CT scan that suggest malignancy include circumferential pleural thickening, pleural nodularity, parietal pleural thickening greater than 1 cm, and involvement of the mediastinal pleura.

A CT scan scoring system [28] has been proposed for distinguishing malignant from benign pleural conditions including parameters such as pleural lesions larger than 1 cm, presence of hepatic metastasis, a pulmonary mass or nodule over 1 cm, pericardial effusion, absence of loculations, and no enlargement of the cardiac silhouette. A score greater than seven out of ten on this scale is indicative of malignancy, with a sensitivity of 88% and a specificity of 94%.

#### 2.1.4. Positron Emission Tomography

The utility of positron emission tomography (PET) in routine MPE diagnosis remains debatable [29]. It has moderate sensitivity and specificity (82% and 74%, respectively) [30]. False negatives can occur in cases of early or slow-progressing disease; on the other hand, conditions like inflammatory pleural involvement, rheumatoid arthritis, and prior pleurodesis procedures can lead to false-positive results [31]. Recently, the TARGET trial [32] aimed to compare PET-CT-guided pleural biopsy to CT-guided biopsy in suspected malignant pleural thickening; the results did not support the routine practice of PET-CT to guide pleural biopsy.

#### 2.1.5. Magnetic Resonance Imaging

Magnetic resonance imaging (MRI) provides higher-quality images of soft tissues compared to CT scans, making it more sensitive for detecting chest-wall and diaphragmatic involvement [33]. However, MRI has lower image quality for lung parenchyma, higher costs and limited accessibility, leading to its limited use in standard diagnostic algorithms. Diffusion-weighted imaging (DWI) is helpful for distinguishing between benign and malignant pleural conditions [34].

#### 2.1.6. Pleural Manometry

Hemodynamic electronic transducers are considered the most accurate and reliable method for assessing intrapleural pressure. Pleural manometry has been shown to be useful in optimizing pleurodesis for patients with MPE [35], and it has been suggested as a method to detect an unexpandable lung after thoracentesis. However, there is no established consensus on specific pressure thresholds to differentiate between normal and unexpandable lungs.

### 2.2. Histopathology

Histopathological examination is essential for diagnosing MPE, as it provides detailed insights into cell types and tissue structures, allowing differentiation between malignant cells and reactive mesothelial cells, as well as providing characterization of malignancy. Several diagnostic techniques are available: pleural fluid cytology, cell block preparation, and pleural biopsy—whether image-guided or thoracoscopic. Recent advances in histopathological techniques and immunohistochemistry have greatly enhanced the ability to detect and characterize MPE [36].

#### 2.2.1. Analysis of Pleural Fluid

Analysis of PF samples includes measuring pH, glucose, proteins, lactate dehydrogenase (LDH), cytology, and microbiology. The following parameters may suggest MPE: LDH higher than 1000 U/L, lymphocyte counts higher than 50–70%, pH below 7.30, and low pleural fluid glucose levels from 30 to 50 mg/dL [37]. Although MPE is typically exudative, according to standard Light criteria [38], it can present as transudative in 5–10% of cases [39]. An optimal volume for diagnosing MPE is around between 40 and 60 mL of fluid [40]. Cytology is the first-line test for diagnosing MPE, with an overall diagnostic accuracy of about 60% [41]. Its sensitivity varies greatly depending on the cancer type, being as low as 6% for mesothelioma and as high as 80% for adenocarcinomas [42]. Low diagnostic performance may be due to tumors not being located on the surface of mesothelial cells where malignant cells can be easily exfoliated into PF. Immunohistochemistry can distinguish between malignant pleural mesothelioma and adenocarcinoma metastases by exposing cells to different antibody panels, each with specific antigens [43]. These markers also help determine the origin of metastasizing adenocarcinoma. Lymphocyte subtype analysis can also identify hematological MPE in specific patient cohorts [44]. A significant challenge in diagnosing MPE by conventional cytology is distinguishing malignant cells from reactive mesothelial cells [45].

The cell-block procedure for fluid processing offers several advantages over conventional cytology, particularly in retaining tissue fragments [46]. An additional benefit of the cell-block method is its ability to preserve antigenicity and cytomorphological features. The increased sensitivity of this approach is due to higher cellularity and better preservation of cellular architecture and the morphological patterns of malignant cells. Moreover, cell-block specimens can be used for immunohistochemistry and special staining, enhancing diagnostic capabilities [47].

#### 2.2.2. Pleural Biopsy

Pleural biopsy remains the gold standard for diagnosing MPE [48]. Due to the limited diagnostic performance of cytology and the lack of standardized protocols for cell block preparation, a pleural biopsy is advised for patients who have negative cytology results [49]. Common percutaneous pleural biopsy techniques include the Abrams, Cope, Vim Silverman, and Tru-Cut biopsies [50]. The latter seems to be more sensitive than the others [51]. Pleural biopsy remains a favored option because it is easier to perform and more cost-effective than more invasive procedures, such as thoracoscopy. The use of imaging techniques like ultrasonography and CT to guide biopsy can further enhance the accuracy of the diagnosis, increasing the sensitivity by up to 80–90% [52]. For MPE with pleural thickening greater than 1 cm, CT-guided pleural biopsy has a similar diagnostic sensitivity to thoracoscopy [53].

#### 2.2.3. Medical Thoracoscopy

Medical thoracoscopy, performed under local anesthesia and conscious sedation, enhances diagnostic accuracy by enabling direct visualization of the affected area and allowing for precise tumor tissue sampling. Medical thoracoscopy is a safe, minimally invasive procedure with few complications and mortality rates, and high diagnostic accuracy, making it suitable for patients who are not surgical candidates or who are at high risk of complications from more invasive procedures like video-assisted thoracoscopic surgery (VATS). The rigid thoracoscope has traditionally been used for these procedures, but a semiflexible thoracoscope has become more popular recently [12]. This semiflexible device offers better maneuverability. However, the smaller working channel of the semiflexible thoracoscope can restrict the size and depth of biopsies. Despite these differences, the diagnostic effectiveness of medical thoracoscopy does not appear to be significantly affected by the choice between rigid and semiflexible thoracoscopes [54]. Forceps and cryoprobe biopsies are comparable and complementary methods used to obtain tissue samples by medical thoracoscopy: the cryoprobe obtains deeper tissue specimens with less crush artifacts, whereas the forceps can obtain larger tissue specimens than the cryoprobe.

## 3. Treatment

**Figure 2:**
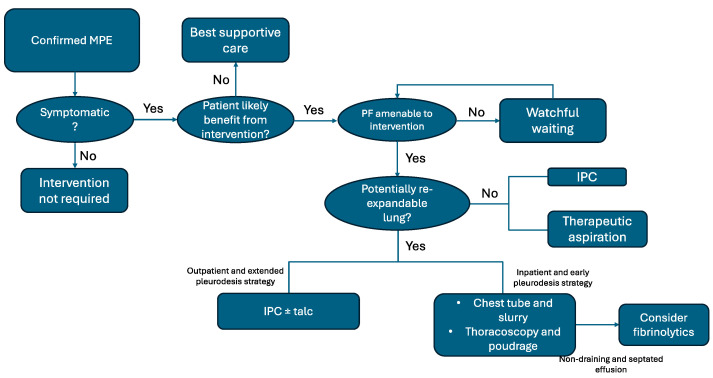
Treatment algorithm in MPE (adapted from [14]).

The primary goal of MPE treatment is symptom relief [14]. Several techniques can be applied to treat symptomatic MPE: repeated therapeutic thoracentesis, thoracic drainage, IPC, talc, or surgery. Over the past decade, several randomized controlled trials have been conducted to explore various aspects of MPE management, including the optimal drain size, different methods for administering talc, the best techniques for achieving pleurodesis, and IPC drainage. Treatment choices should be individualized based on clinical factors and patient preferences. Tools such as the PROMISE and LENT scores [9,10], can help guide treatment decisions. Evaluating whether the lung can fully re-expand and ruling out a ‘trapped lung’ are crucial first steps in determining the appropriate treatment strategy (Figure 2).

### 3.1. Thoracentesis

Therapeutic thoracentesis is indicated for all patients with MPE affecting 50% of the hemithorax and causing dyspnea. Thoracentesis is not intended to prevent re-accumulation of fluid or to facilitate ongoing drainage; instead, the procedure is primarily used to verify the presence of fluid and assess lung re-expansion after the PF has been removed. Although not considered a definitive intervention, for patients with a limited life expectancy (days to weeks), repeated thoracentesis, of up to 1.5 L per day, may be indicated to alleviate symptoms. Volumes over 1.5 L might lead to re-expansion pulmonary edema [55].

### 3.2. Pleurodesis

Pleurodesis aims to create an inflammatory response within the pleural cavity leading to obliteration and symphysis between the parietal and visceral pleura, thus preventing fluid accumulation [56]. It has been shown to improve dyspnea, to increase survival, and to reduce hospital stays and future interventions [57]. However, pleurodesis is not recommended if there is a trapped lung or multiple pleural septa, as successful pleural adhesion is unlikely. Pleurodesis can be performed through either mechanical abrasion or by introducing chemical agents like talc, antibiotics (doxycycline, tetracycline), antiseptics (povidone-iodine, silver nitrate), microorganisms (Streptococcus pyogenes, Corynebacterium parvum), chemotherapeutics (bleomycin, doxorubicin, mitomycin), and autologous blood [58]. The optimal sclerosing agent for pleurodesis is not yet established, but talc is widely used due to its availability and cost-effectiveness [59]. It can be administered via aerosol through a thoracoscope (talc poudrage) or as a suspension through an intercostal tube (talc slurry). A recent meta-analysis suggests that talc insufflation is the most effective pleurodesis method for preventing fluid re-accumulation [60]. Another promising sclerosing agent is Viscum [61,62], a liquid derived from European mistletoe, which not only produces inflammation but also stimulates the immune system providing a potential anti-tumoral effect.

Hospitalization is required for pleurodesis, whether using a chest tube or thoracoscope. Chest tube insufflation can be performed at the bedside with analgesia, while thoracoscopy requires general anesthesia or conscious sedation. Randomized clinical trials have not demonstrated a clear advantage of one technique over the other [63,64]. The recently concluded TAPPS trial showed no significant differences in pleurodesis failure rates at 90 days after randomization between the thoracoscopic insufflation method and the bedside chest drain talc slurry procedure [65].

Pleurodesis success rates vary from 53% up to 91% [63,66]. When using talc, the use of large-particle talc is recommended to prevent acute respiratory distress syndrome. Other potential complications include fever and chest pain from intrapleural talc.

The size of the drainage tube used for pleurodesis is another debated topic. The TIME-1 trial [67] suggests that large-bore tubes are more effective. Smaller tubes, however, are more comfortable for patients. The same study indicated that using nonsteroidal anti-inflammatory drugs to control pain does not affect pleurodesis outcomes. Although no clinical data suggest corticosteroids negatively impact pleurodesis efficacy, their use should be minimized.

The recent OPTIMUM trial [68] found no differences between an outpatient pathway using IPC with or without talc pleurodesis and an inpatient pathway using chest-tube talc pleurodesis, in terms of quality-of-life improvement.

The TIME-3 trial [69] investigated whether using urokinase to break down septations before administering talc could affect the time to pleurodesis failure and changes in dyspnea scores. The study found no difference in the primary outcomes between the urokinase and placebo groups, even though radiological improvements were observed in the urokinase group.

### 3.3. Indwelling Pleural Catheter

IPCs are silicone tubes inserted percutaneously with a one-way valve, allowing intermittent fluid drainage to maintain lung expansion. IPCs aim to control symptoms without hospitalization and are as effective as pleurodesis as a first-line treatment for MPE [70]. In cases of unsuitable pleurodesis, recurrent effusions post-pleurodesis, or in the presence of a trapped lung, IPC insertion is considered a standard approach. A systematic review showed that IPCs improved symptoms in 95% of cases and achieved spontaneous pleurodesis in 46–70% of patients with full lung expansion [71]. Although IPCs reduce hospital stays and are less invasive than pleurodesis, they require more frequent care and are associated with higher complication rates [72]. Bertolaccini and colleagues [73] highlighted the lower complication rates associated with the early insertion of IPCs compared to repeated needle thoracentesis. The TIME-2 trial [74] did not find significant differences between talc pleurodesis and IPC in relieving dyspnea, whereas the AMPLE trial [75] found that patients undergoing IPC positioning had shorter hospital stays compared to those undergoing talc pleurodesis. Both the AMPLE2 and ASAP trials also showed that patients who underwent IPC placement with aggressive drainage had higher rates of autopleurodesis and achieved it faster than those who had IPCs with alternate-day drainage [76,77]. The IPC-PLUS trial [78] compared patients with symptomatic MPE undergoing IPC followed by talc-slurry or placebo-slurry: the results showed nearly double the success rate in the talc group compared to the placebo group.

The SWIFT trial [79], which evaluated pleurodesis success rates using silver nitrate-coated IPCs versus standard IPCs, did not support the use or further evaluation of the former against the latter.

### 3.4. Surgery

VATS is an important procedure used for both diagnosing and treating pleural diseases, with wide-ranging applications [80]. The diagnostic accuracy is high, with 95% sensitivity for malignancies, and its effectiveness in preventing effusion recurrence by chemical pleurodesis is well established [81]. VATS is safe, with low mortality and morbidity rates. It is generally performed in the operating room, with the patient under general anesthesia and intubated. While both medical thoracoscopy and VATS can be employed for pleural biopsies and pleurodesis, VATS offers better access for draining loculated effusions surrounded by thick fibrous tissue and can be converted into an open thoracotomy if necessary [82]. The mean operative time required for parietal pleural biopsy is less than 20 min [83]. The video-assisted approach with single-lung ventilation provides optimal conditions for surgery by minimizing lung injury, fully aspirating pleural fluid, breaking adhesions, ensuring proper talc distribution, and promoting full lung re-expansion. Surgical pleurectomy is rarely used for MPE management due to the potential for high perioperative mortality and a decline in quality of life. For mesothelioma-related MPE, talc pleurodesis is generally preferred over surgery due to lower complication rates and shorter hospital stays [84].

## 4. Future Perspectives

Several clinical trials are underway to further explore MPE management strategies. The MESOTRAP [85] trial compares VATS partial pleurectomy/decortication and IPC in patients with trapped lung and pleural mesothelioma. The AMPLE3 [86] trial is the first randomized study to compare the effectiveness of IPC (with or without talc) versus VATS pleurodesis in patients eligible for surgery. The TACTIC trial [87] compares the combination of thoracoscopic talc poudrage with concomitant IPC positioning against thoracoscopic talc poudrage alone.

## 5. Conclusions

The diagnosis of MPE has seen significant advancements and will continue to improve with the development of more effective molecular techniques. However, because cytology alone has limited sensitivity and, even when positive, may not provide comprehensive information for treatment, biopsy remains a crucial component in evaluating suspected MPE.

The optimal management of MPE remains uncertain and requires a multidisciplinary approach. Initial steps involve less invasive diagnostic tests, followed by more invasive procedures if necessary, considering patient preferences, functional status, life expectancy, tumor type, and medical team expertise. Current evidence supports both talc pleurodesis and IPC for symptom relief and fluid reduction, but further research is needed to refine these techniques and provide personalized care based on specific patient characteristics. In the coming years, combined diagnostic and therapeutic approaches that facilitate near-total outpatient management of MPE are likely to gain popularity. However, these innovations will need to be supported by robust evidence to evaluate their effectiveness, benefits, and risks for patients.

## Data Availability

All data are available upon request.
